# Risk stratification for melanoma

**DOI:** 10.18632/oncotarget.26755

**Published:** 2019-03-08

**Authors:** Catherine M. Olsen, David C. Whiteman

**Affiliations:** Cancer Control Group, QIMR Berghofer Medical Research Institute, Herston, Brisbane, Australia

**Keywords:** melanoma, risk stratification

The incidence of cutaneous melanoma has increased steadily since the 1960's in most susceptible populations across the world [1]. Control strategies include primary prevention to reduce incidence, and early detection to reduce morbidity and mortality. Population-based screening is generally not recommended by medical authorities due to a paucity of evidence that potential benefits outweigh harms [2]. The only country where a population-based screening program has been implemented is Germany, and despite early indications of a reduction in mortality, the decline was not sustained at 5-years post-initiation [3].

One alternative to population-screening to detect melanoma at an early stage is to adopt a targeted screening approach whereby ‘high risk’ sub-groups are identified for entry to a screening program. For example, screening among people with a family history and/or personal history of melanoma and/or dysplastic nevus syndrome has been shown to be effective and cost-efficient [4, 5]. Most people who develop melanoma do not have these characteristics however, and so the challenge has been to identify other ‘high risk’ groups who do not have these phenotypic traits. To date, a number of risk algorithms have been developed to assist with this task [6, 7].

We recently developed and published a risk prediction tool for melanoma developed in a large, population-based, prospective study that was purpose designed to examine melanoma and skin cancer outcomes [8]. All candidate predictive factors (n=28) were self-reported on the baseline survey; these were selected *a priori* from the literature and also from clinician input. We examined two outcomes: invasive melanoma and all melanoma (i.e. both invasive and *in situ* melanoma). We used forwards and backwards stepwise regression techniques to derive a parsimonious model for invasive melanoma, which included seven terms: age, sex, tanning ability, number of nevi at age 21, hair color, number of previous non-surgical treatments for actinic lesions, and sunscreen use. The model for ‘all melanoma’ included these seven terms and an additional five, namely family history of melanoma, ethnicity, number of excisions for skin cancers, history of skin checks by a doctor and private health insurance. Both models showed high discrimination in the development (AUC 0.79 and 0.74 for invasive and ‘all melanoma’, respectively) and validation samples (AUC 0.69 and 0.72). Importantly, we also found that there was low concordance between a person's self-assessed risk and their risk as determined by the prediction tool. In other words, most individuals have poor ability to assess their future risk of melanoma.

We assessed the sensitivity and specificity of the models within deciles of predicted risk; these were maximized in the seventh decile of the risk distribution with a sensitivity of 74.2% and specificity of 60.7%. If this level of risk was used as the threshold for entry into a screening program, then 34 people would need to be screened to detect one melanoma within the next 3.4 years. We also conducted decision curve analyses to evaluate the clinical utility of the model across the full range of the risk distribution; the model performed better than the “screen all” or “screen none” approaches when the risk probability was 0.3-8.9%.

The risk calculator was made available online following publication (https://publications.qimrberghofer.edu.au/Custom/QSkinMelanomaRisk/), and has been used by over 200,000 people since March 2018. Risk information is returned to users in five categories (relative to others of the same age group and sex: very much above average; above average; average; below average; very much below average), and general advice is communicated depending on risk level. De-identified data on risk factors has been collected for all completed sessions. Feedback from members of the community who have used the calculator has been very positive, and several large groups subsequently visited the Institute to learn more about it, and about melanoma research in general. Over 80,000 people have indicated that they are willing to take part in future research studies conducted at the Institute. The research outcome (risk stratification tool) aligns well with the needs of Australians, who are at high risk of melanoma due to high ambient ultraviolet radiation levels, and has provided a mechanism for community engagement that has resulted in a valuable resource for future research. We believe that this type of community engagement may lead to improvements in health promotion and disease prevention as well as achieve the aims of present and future health research activities.

In summary, we have developed a risk stratification tool for melanoma that is suitable for use in the Australian population. The items included in the model can be easily determined through self-report, either in a clinical setting or remotely. We believe the tool will aid clinicians and patients to quantify risk and to assist clinical decision-making in regards to personalised surveillance. It may also promote awareness of the factors that contribute to melanoma risk. In our future research, we aim to validate the tool in clinical settings. We also have genomic data for a large proportion of the cohort, and will examine the impact of including this information on the performance of the model. Finally, it remains to be determined how well this tool, developed in Australia for Australian populations, performs when transferred to other settings.
